# Evaluation of intra‐fractional target displacement by patient motion during a single‐isocenter multi‐target stereotactic radiation therapy for brain metastases

**DOI:** 10.1002/acm2.70219

**Published:** 2025-08-21

**Authors:** Ryota Yamada, Takaaki Yoshimura, Ryo Murata, Kentaro Nishioka, Takashi Mori, Fuki Koizumi, Yoshihiro Fujita, Shuhei Takahashi, Takahiro Hattori, Takahiro Kanehira, Kohei Yokokawa, Rie Yamazaki, Kenji Horita, Hiroshi Tamura, Yamato Wakabayashi, Yuta Ichiu, Takayuki Hashimoto, Hidefumi Aoyama

**Affiliations:** ^1^ Department of Radiation Technology Hokkaido University Hospital Sapporo Japan; ^2^ Department of Health Sciences and Technology Faculty of Health Sciences Hokkaido University Sapporo Japan; ^3^ Department of Medical Physics Hokkaido University Hospital Sapporo Japan; ^4^ Global Center for Biomedical Science and Engineering Faculty of Medicine Hokkaido University Sapporo Japan; ^5^ Graduate School of Biomedical Science and Engineering Hokkaido University Sapporo Japan; ^6^ Department of Radiation Oncology Hokkaido University Hospital Sapporo Japan; ^7^ Department of Radiation Oncology Faculty of Medicine Hokkaido University Sapporo Japan

**Keywords:** brain metastases, fractionated stereotactic radiosurgery, intra fractional patient motion, mouthpiece, single‐isocenter multi‐target SRT

## Abstract

**Background:**

Single‐isocenter multi‐target volumetric modulated arc therapy (SIMT‐VMAT) has been implemented widely in fractionated stereotactic radiosurgery (fSRS) to treat brain metastases. The impact of rotational intra‐fractional patient motion (IFPM) is influenced by the distance between the geometric target's center and the isocenter (DTI).

**Purpose:**

We hypothesized that IFPM's impact on each target would increase with greater DTI during fSRS. Therefore, we aimed to estimate the intra‐fractional target displacement (IFTD), which represents each target's displacement caused by translational and rotational components of IFPM.

**Methods:**

In this study, we involved 35 patients with 2–13 brain metastases, all of whom had previously undergone SIMT‐VMAT fSRS. All patients were immobilized using a clamshell‐style device, with 28 using a biteplate. Cone beam computed tomography (CBCT) images were obtained at the same imaging center before and after treatment. The IFPM was calculated using both CBCT datasets. The IFTD was determined by comparing the planned target coordinates with the actual coordinates while factoring in IFPM.

**Results:**

We evaluated 136 targets. The mean IFTD was 0.38 mm (95% confidence interval [CI]: 0.37–0.40 mm) with the biteplate and 0.65 mm (95% CI: 0.59–0.71 mm) without it. A very weak positive correlation was observed between DTI and IFTD despite the immobilization method. This correlation indicates that the distance dependence of IFTD is nearly negligible.

**Conclusion:**

The findings showed that the impact of IFPM on each target demonstrated minimal dependence on the DTI. Displacement was relatively consistent regardless of the target location. In addition, the use of a biteplate was suggested to potentially reduce these effects.

## 1 INTRODUCTION

According to various studies, brain metastases are common neurologic cancer complications and the most prevalent type of brain tumor, occurring in approximately 20%–40% of adult patients with cancer.^1^, ^2^ Historically, whole‐brain radiotherapy (WBRT) was the standard treatment for multiple brain metastases. Recently, the treatment shifted to stereotactic irradiation (STI), including single‐session stereotactic radiosurgery (SRS) and fractionated SRS (fSRS), to minimize neurocognitive deterioration and preserve the patient's quality of life (QOL).^3^, ^4^

Currently, there are various principal modalities for delivering STI, including Gamma Knife and linear accelerator (LINAC)‐based approaches. Traditionally, LINAC‐based SRS or fSRS required a separate isocenter for each treated target, requiring approximately 20 min to treat each lesion.^5^ Amaya et al. reported that in LINAC‐based SRS, the average patient table time was 30 min using the single‐isocenter technique, whereas, with the multi‐isocenter technique, it was approximately five times longer, at 144 min.^6^ In addition, with increasing isocenters, more time is required for dose verification and treatment planning. During treatment, multiple setups and repeated image guidance with CBCT or similar modalities are needed, which not only raises the risk of errors but increases the workload for therapists substantially.

Minimizing treatment time is critical owing to patient movement, which increases during prolonged irradiation, especially in fSRS. Currently, single‐isocenter multi‐target volumetric arc therapy (SIMT‐VMAT) is a proposed faster treatment option for multiple brain metastases.^7^ In addition, Ikawa et al. reported that LINAC‐based fSRS for brain metastases, including large and multiple lesions, is safe with a low incidence of immediate side effects.^8^

STI requires highly accurate treatment delivery, which was traditionally achieved using thermoplastic masks or headframes; however, the latter had several potential disadvantages. These disadvantages include pin placement discomfort, the need to account for prior cranial surgeries, rare cases of bleeding or infection, additional monitoring with conscious sedation, and occasional frame slippage.^9^, ^10^ Recent advancements in highly reproducible patient positioning techniques and image‐guided localization have mitigated uncertainties during LINAC‐based STI treatments. Bolten JH et al. reported that phantom tests showed the capability of cone beam computed tomography (CBCT) and other image‐guidance techniques used in LINAC‐based STI to achieve highly accurate patient positioning within a submillimeter range.^11^ In addition, Ohira et al. reported that the intra‐fractional motion error during STI was <1 mm for the open‐ and full‐face clamshell‐style immobilization systems. This finding revealed that either device can be used effectively for patient immobilization.^12^

Despite using image guidance and a six‐degree‐of‐freedom couch to minimize uncertainty during treatment, slight residual setup errors persist.^13^ Considering that a single isocenter is used in SIMT‐VMAT fSRS for multiple brain metastases, each target's displacement varies based on its distance from the isocenter. Specifically, the rotational components have a greater effect with increased distance from the isocenter.^14^, ^15^, ^16^ Roper et al. suggested that the rotational error should be ≤0.5° due to the target dose coverage depending on the distance between the target and the treatment isocenter.^14^ Prentou et al. suggested that limiting the lesion to an isocenter distance of ≤4 cm by introducing an additional isocenter partly mitigate severe target underdosage.^15^ Nakano et al. recommended excluding targets > 7.6 cm from the isocenter using the SIMT‐VMAT fSRS technique to meet tolerance values for all gross tumor volumes (GTVs).^16^

In SIMT‐VMAT fSRS, rotational effects involving intra‐fractional patient motion (IFPM) are more effective for metastases farther from the isocenter. To assess IFPM‐related uncertainty, residual rotational and translational errors should be calculated per metastasis. Furthermore, to mitigate IFPM, evaluating the fixation accuracy of clamshell immobilization devices and the added robustness provided by biteplates is essential. Therefore, a comprehensive analysis of IFPM‐induced target displacement in six degrees of freedom, and its correlation with the isocenter distance and fixation stability, is required.

We hypothesized that IFPM during SIMT‐VMAT fSRS may cause differential displacement of individual brain metastases based on their distance from the isocenter. Therefore, if such a distance dependency exists, characterizing it would be important, as it may indicate the need to individualize margins accounting for IFPM according to the intracranial location of each metastasis when treating multiple brain tumors.

In this study, we aimed to quantitatively evaluate the relationship between IFPM‐induced intra‐fractional target displacement (IFTD) and the distance between the target's geometric center and the isocenter (DTI) during SIMT‐VMAT fSRS. IFTD was evaluated across six degrees of freedom. In addition, we examined whether using a biteplate reduces IFPM.

## 2 METHODS

### 2.1 Patient data and image acquisition

**FIGURE 1:**
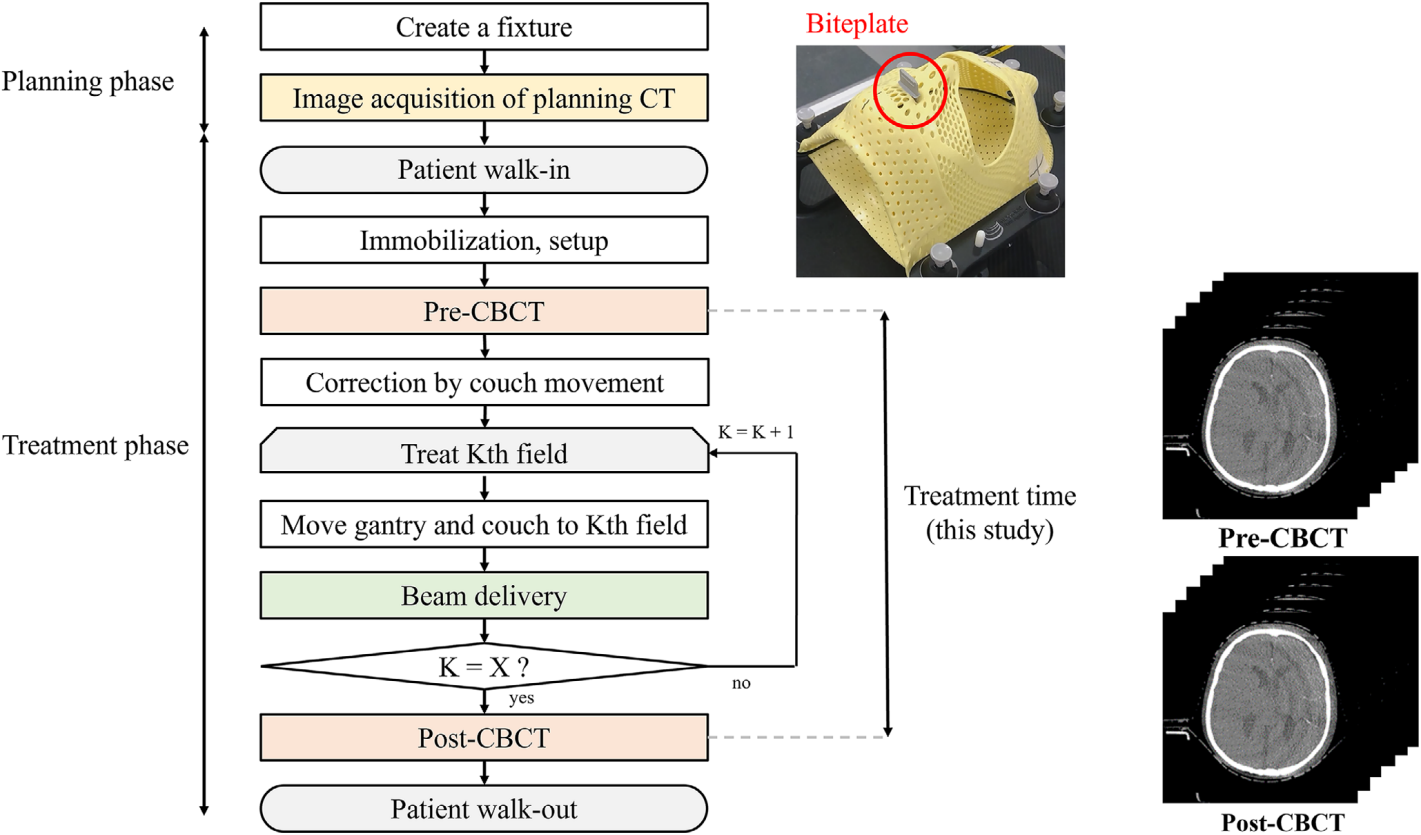
Flowchart illustrating the SIMT‐VMAT fSRS process related to image acquisition in this study. SIMT‐VMAT, single‐isocenter multi‐target volumetric arc therapy; fSRS, fractionated stereotactic radiosurgery. “K” represents the index number for each treatment field, “X” represents the total number of treatment fields.

In this retrospective study, 35 patients with multiple brain metastases who had previously undergone SIMT‐VMAT fSRS at our institution from March 2021 to April 2022 were included. Table 1 shows the patient characteristics. The Institutional Review Board approved this study.
All patients were immobilized using the clamshell style of the QFix Encompass SRS immobilization system (CQ Medical, Avondale, PA). Of these patients, 28 were fitted with a biteplate within the shell, while seven were not. The primary reason for avoiding the biteplate was the presence of complete dentures. No significant differences were observed among the 35 patients regarding their consciousness level, respiratory status, or cognitive understanding, which is vital for undergoing fSRS. We conducted a computed tomography (CT) scan using the SOMATOM Confidence RT Pro (Siemens Healthineers, Forchheim, Germany). The images were reconstructed with a slice thickness of 1.0 mm and exported to the Eclipse TPS version 15.6.8 (Varian Medical Systems, Palo Alto, CA). CT images, with or without contrast, were acquired using a tube voltage of 120 kVp. Gadolinium‐enhanced three‐dimensional (3D) T1‐weighted magnetic resonance (MR) images were used for radiation treatment planning. These images were acquired within 2 weeks of the treatment planning CT and were rigidly registered to it using the Eclipse TPS for target delineation. T1‐weighted MR imaging was performed with a 3D fast spin‐echo sequence on 3T MR scanners: Elition (Philips Healthcare, Best, the Netherlands), Discovery 750 W (GE Healthcare, USA), and TRILLIUM OVAL (Hitachi, Ltd, Tokyo, Japan).
At the start of each treatment, CBCT images were used to reposition the patient along six axes to align the isocenter coordinates with those of the treatment plan. A 360‐degree full scan of the CBCT images was acquired using a tube voltage and current (125 kVp and 720 mAs, respectively), with a LINAC‐mounted onboard imager. The image reconstruction parameters were a slice thickness of 2.0 mm, a matrix size of 512 × 512, and a field of view of 25.2 cm. As shown in Figure 1, CBCT images were acquired pre‐ and post‐treatment within the same imaging center to assess IFPM.

**TABLE 1:** Characteristics of this study's patients.

								Range		
					*N*	(%)	Median	Min	–	Max
Number of patients					35				–	
Sex	Male				17	48.6%			–	
	Female				18	51.4%			–	
Age							69	49	–	83
Biteplate	With				28	80.0%			–	
	Without				7	20.0%			–	
Number of targets	Total				136				–	
	Per patient						3	2	–	13
	DTI (mm)	0	–	19	10	7.4%			–	
		20	–	29	11	8.1%			–	
		30	–	39	30	22.1%			–	
		40	–	49	27	19.9%			–	
		50	–	59	23	16.9%			–	
		60	–	69	25	18.4%			–	
		70	–	79	7	5.1%			–	
		80	–	89	3	2.2%			–	

Abbreivation: DTI, distance between target and isocenter.

### 2.2 Treatment planning and dose delivery

The Hyperarc software (Varian Medical Systems, Palo Alto, CA) was used to treat all patients. This supported the automatic optimization and delivery of non‐coplanar SIMT‐VMAT using the Varian TrueBeam version 2.7 or higher, combined with the Eclipse TPS version 15.1 or higher (Varian Medical Systems, Palo Alto, CA).^8^, ^17^, ^18^ Furthermore, all images were loaded into the Eclipse TPS, where the GTVs and visible lesions on the CT and MR images were contoured by radiation oncologists. The treatment isocenter was positioned approximately equidistant from each GTV. The SIMT‐VMAT fSRS plans were generated using four non‐coplanar arcs (couch angles [deg]: 0, 45, 270, and 315) employing auto couch movement to deliver 24 or 32 Gy based on the TrueBeam LINAC (Varian Medical Systems, Palo Alto, CA). A dose of 32 Gy in four fractions was prescribed following the treatment planning guidelines of the Japanese Society for Radiation Oncology. However, the dose was reduced to 24 Gy in four fractions if the target was adjacent to the optic nerve or brainstem.^19^ For each plan, 6‐MV photon flattening filter‐free beams at 1400 monitor units per min were used (MU/min).

Image guidance is essential for safe and accurate treatment beam delivery in SIMT‐VMAT fSRS to correct both translational and rotational directions.^14^ The planning CT and CBCT images were registered automatically using six‐dimensional rigid bony registration (anterior‐posterior [AP], superior‐inferior [SI], left–right [LR], pitch, roll, and yaw). We applied the full automation method to the SIMT‐VMAT fSRS process. The patient position was corrected before beam delivery if the deviation exceeded 0.5 mm or 0.5 degrees. Treatments were managed with ARIA ver. 13.6 (Varian Medical Systems, Palo Alto, CA).

In this study, we used the machine log data to analyze the patient treatment process flow. Using previous reports on treatment process flow analysis in radiation therapy,^20^, ^21^, ^22^, ^23^ the treatment time of SIMT‐VMAT fSRS was defined as the time between pre‐ and post‐CBCT.

Definitions of target's displacement by IFPM

**FIGURE 2:**
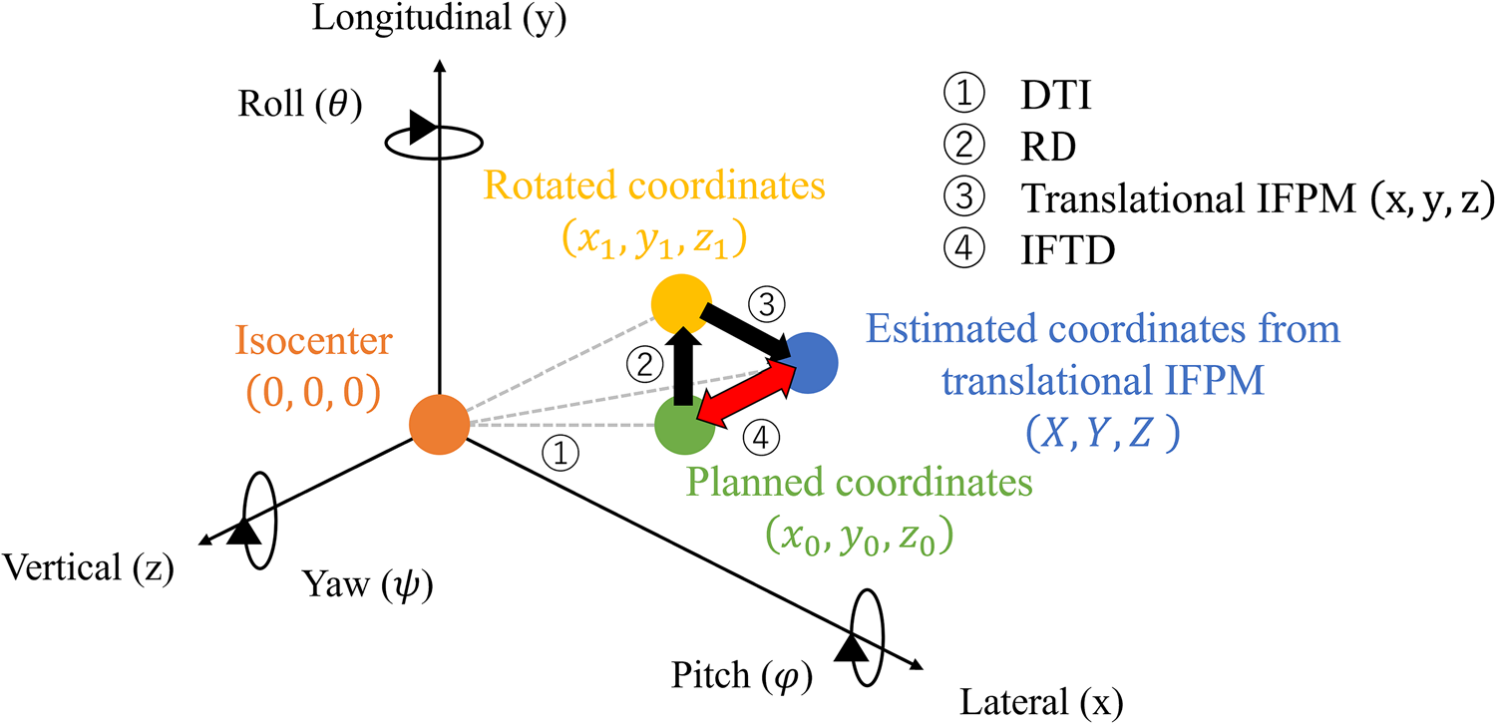
Parameter definitions in this study. DTI, distance between target and isocenter; RD, rotational displacement; IFPM, intra‐fractional patient motion; and IFTD: intra‐fractional target displacement.

The treatment time was defined as the duration between the acquisition time of pre‐ and post‐CBCT (Figure 1). As shown in Figure 2, the DTI was defined as follows (Equation 1):

(1) $$DTI\;\;\left[mm\right]=\sqrt{{x_{0}}^{2}+{y_{0}}^{2}+{z_{0}}^{2}}$$

Where **x₀**, **y₀**, and **z₀** represent the center of gravity coordinates of each GTV in the treatment planning CT. IFPM was defined as the amount of couch shift between pre‐ and post‐CBCT, divided into translational (x, y, z) and rotational (φ, θ, ψ) directions. Each estimated target position (X, Y, Z) was defined as the geometric target's center, calculated in the treatment planning system using the isocenter as the origin (Equation 2).

(2) $$\begin{array}{ccc} \;\left(\begin{array}{c} X\\ Y\\ Z \end{array}\right) & = & \left(\begin{array}{ccc} \cos\psi & -\sin\psi & 0\\ \sin\psi & \cos\psi & 0\\ 0 & 0 & 1 \end{array}\right)\;\left(\begin{array}{ccc} \cos\theta & 0 & \sin\theta \\ 0 & 1 & 0\\ -\sin\theta & 0 & \cos\theta \end{array}\right)\\ & & \times \left(\begin{array}{ccc} 1 & 0 & 0\\ 0 & \cos\varphi & \sin\varphi \\ 0 & \sin\varphi & -\cos\varphi \end{array}\right)\;\left(\begin{array}{c} x_{0}\\ y_{0}\\ z_{0} \end{array}\right)+\left(\begin{array}{c} x\\ y\\ z \end{array}\right) \end{array}$$

Translational IFPM was defined as follows (Equation 3):

(3) $$Translational\;IFPM\;\;\left[mm\right]=\sqrt{x^{2}+y^{2}+z^{2}}$$

In addition, 3D rotational setup error was defined as rotational displacement (RD) as follows (Equation 4):

(4) $$RD\;\;\left[mm\right]=\sqrt{{\left(x_{1}-x_{0}\right)}^{2}+{\left(y_{1}-y_{0}\right)}^{2}+{\left(z_{1}-z_{0}\right)}^{2}}$$

where, $x_{1}$, $y_{1}$, and $z_{1}$ are the rotated coordinates. Finally, IFTD was defined as follows (Equation 5):

(5) $$IFTD\;\;\left[mm\right]=\sqrt{{\left(X-x_{0}\right)}^{2}+{\left(Y-y_{0}\right)}^{2}+{\left(Z-z_{0}\right)}^{2}}$$

An in‐house Python script was developed to perform an automated calculation of each indication.

### 2.3 Evaluation of Couch Rotation Accuracy Using Phantom and Off‐Isocenter Winston‐Lutz (WL) Tests

All treatments were performed using a fully automated system with continuous couch rotation throughout irradiation. Pre‐ and post‐treatment CBCT‐based corrections may not fully capture mechanical uncertainties such as couch walkout during rotation., A head CT phantom (Kyoto Kagaku Co., Ltd., Kyoto, Japan) was immobilized using the same fixation devices as for the patients to evaluate positioning accuracy. CBCT images were acquired at 0°, 45°, 270°, and 315° couch angles, and this procedure was repeated over four consecutive days.

In addition, an off‐isocenter WL test was conducted to evaluate couch rotation uncertainty at non‐zero angles. We used a WL Pointer Phantom Kit with 3D Adjustable Holder (Standard Imaging, Inc., Middleton, WI, USA), with a central ball bearing (BB) marker shifted 8 cm along the Y‐axis to simulate a peripheral target. Electronic portal imaging device images were acquired while rotating the couch from 270° to 90° in 15° increments with the gantry fixed at 0°. Subsequently, using a custom Python script, we calculated the maximum deviation between the BB center and the radiation field center at each angle to quantify the table walkout radius and couch rotation axis offset.

### 2.4 Statistical analysis

Spearman's rank correlation coefficient (r_s_) was used to derive the correlation between RD and IFTD with DTI and between treatment time and IFTD, respectively. In addition, the corresponding p values for r_s_ were calculated. The correlation coefficient r_s_ was categorized as follows: very weak (r_s_ ≤ 0.2), weak (0.2 < r_s_ ≤ 0.4), moderate (0.4 < r_s_ ≤ 0.7), and strong (r_s_ > 0.7). Mann–Whitney's U test was used to compare the biteplate's efficiency. Statistical significance was set at p < 0.05. All statistical analyses were conducted using JMP Pro 16 (SAS Institute Inc., Cary, NC).

## 3 RESULTS

### 3.1 Treatment Time Analysis

**FIGURE 3:**
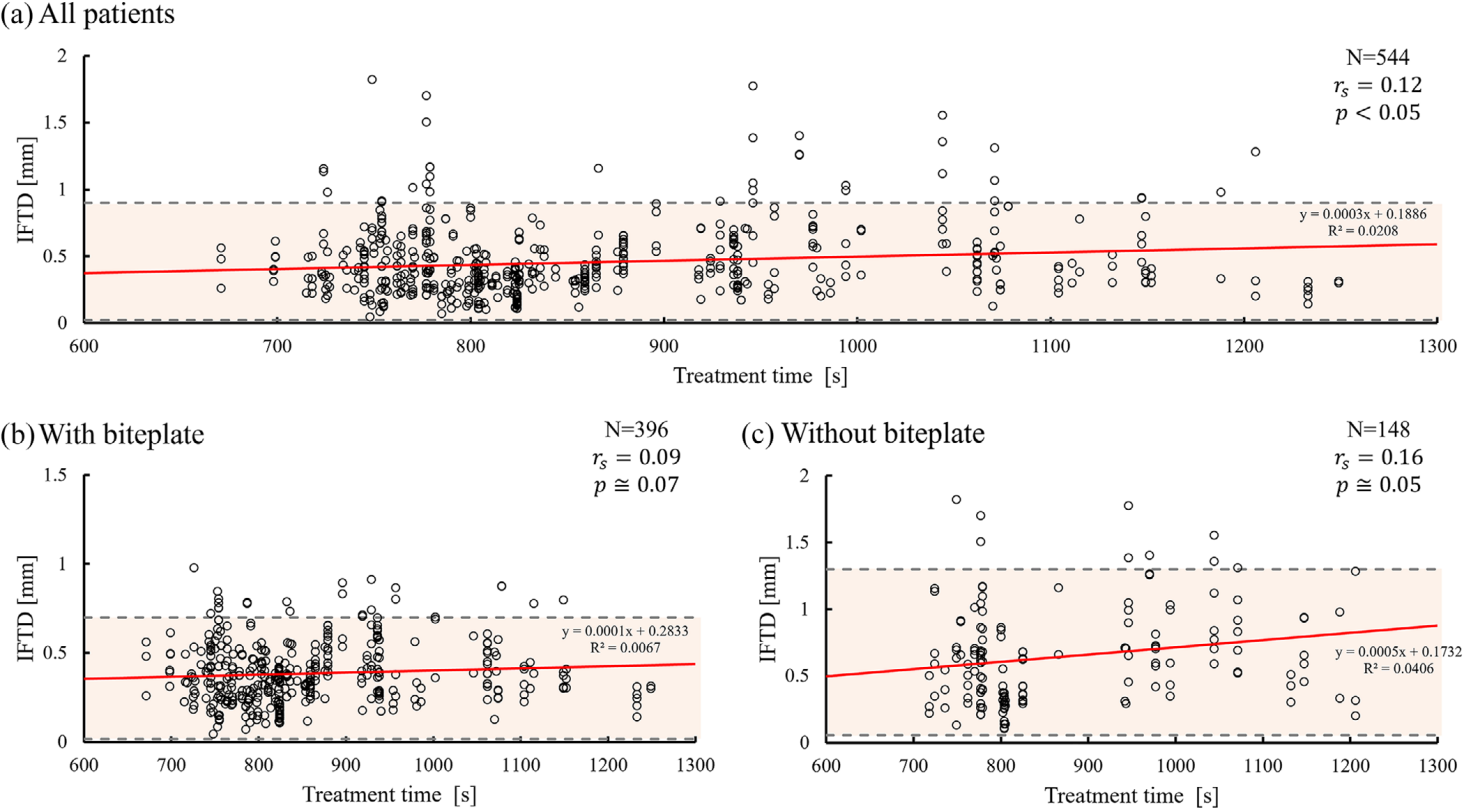
Correlation between treatment time and intra‐fractional target displacement (IFTD). A) All patients. B) with biteplate, and C) without biteplate. The red line represents the linear approximation, while the gray dotted line indicates the 95% confidence interval (CI). IFTD, intra‐fractional target displacement; N, number of patients; and r_s_: Spearman's rank correlation coefficient.

We evaluated 136 targets in 35 patients. The mean treatment time for SIMT‐VMAT fSRS was 861.8 s (95% confidence interval [CI]: 851.4 – 872.3 s). Figure 3 shows a very weak correlation between treatment time and IFTD, indicating that the treatment time for SIMT‐VMAT fSRS does not impact IFTD significantly, regardless of the biteplate use.

### 3.2 IFPM Evaluation with and without Biteplate

**FIGURE 4:**
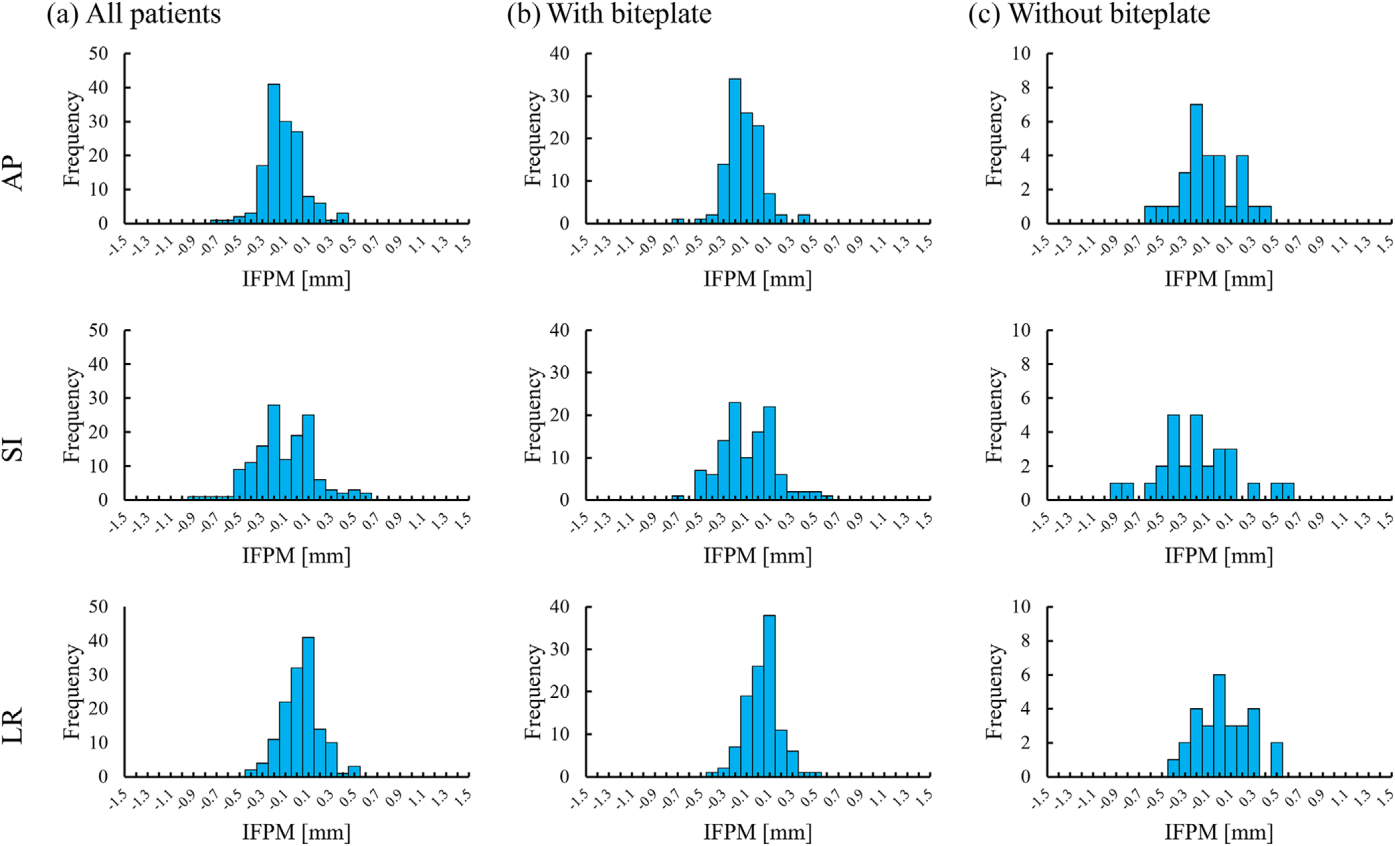
Histograms of IFPM along the translational axes: AP, SI, and LR. IFPM, intra‐fractional patient motion; AP, anterior‐posterior; SI, superior‐inferior; and LR, left‐right.

**FIGURE 5:**
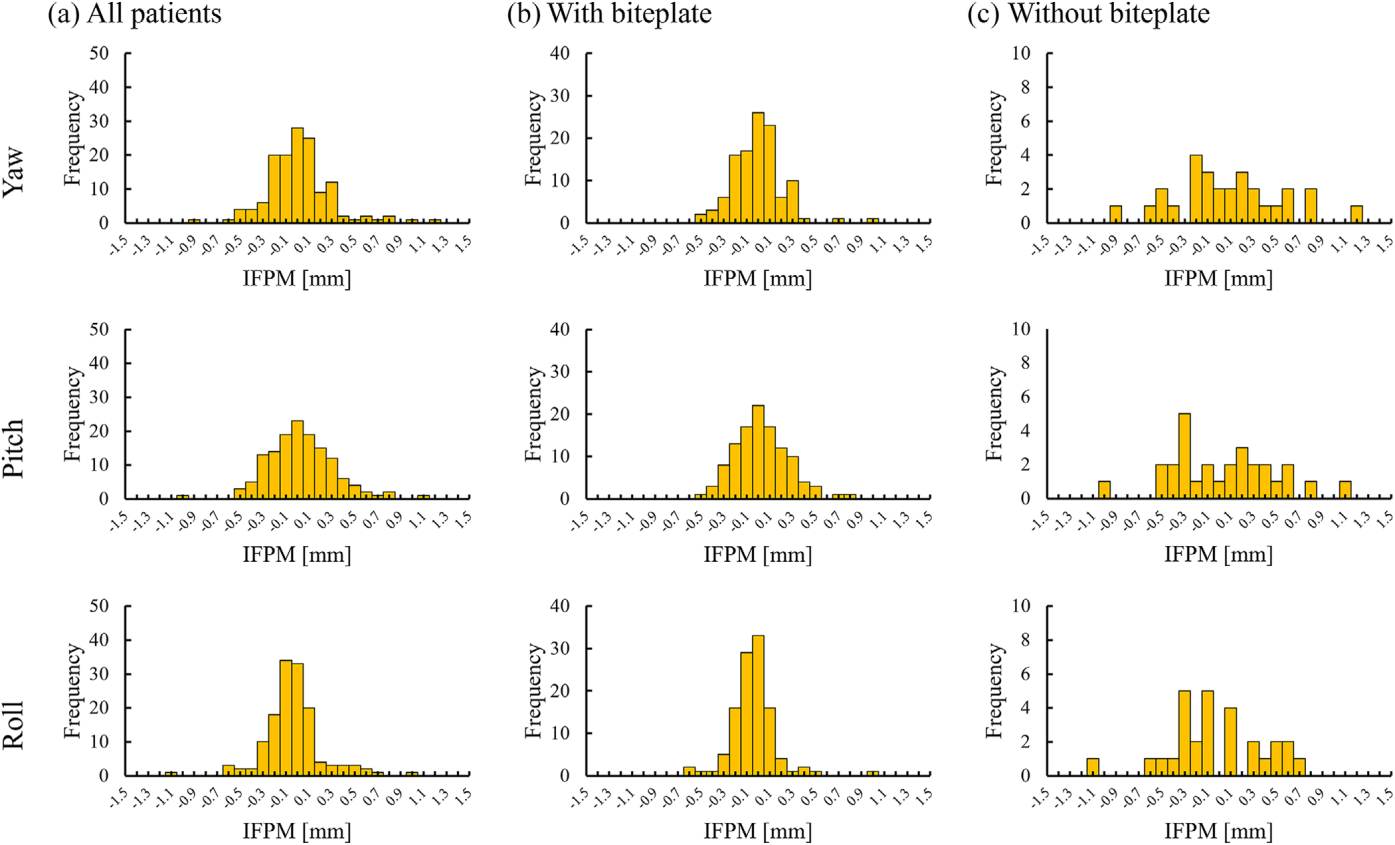
Histograms of IFPM along the rotational axes: yaw, pitch, and roll. IFPM, intra‐fractional patient motion.

In the evaluation of IFPM for each axis, the mean IFPM with the biteplate was ‐0.12 mm (95% CI: ‐0.15 to ‐0.09 mm) in the AP direction, ‐0.09 mm (95% CI: ‐0.13 to ‐0.04 mm) in the SI direction, 0.04 mm (95% CI: 0.01–0.07 mm) in the LR direction, 0.00° (95% CI: ‐0.04–0.04°) in Yaw, 0.03° (95% CI: ‐0.01–0.07°) in Pitch, and ‐0.04° (95% CI: ‐0.08—0.01°) in Roll. However, the mean IFPM without the biteplate was ‐0.09 mm (95% CI: ‐0.19 – 0.00 mm) in AP, ‐0.19 mm (95% CI: ‐0.33 to ‐0.06 mm) in SI, 0.04 mm (95% CI: ‐0.06–0.13 mm) in LR, 0.08° (95% CI: ‐0.10–0.26°) in Yaw, 0.04° (95% CI: ‐0.14–0.22°) in Pitch, and ‐0.03° (95% CI: ‐0.19–0.14°) in Roll. Figures 4 and 5 display IFPM's histogram along the translational and rotational axes, respectively. These figures demonstrate the distribution of IFPM values for all patients, with and without the biteplate, for both translational and rotational components. In evaluating translational IFPM, the mean translational IFPM was 0.32 mm (95% CI: 0.03–0.67 mm) with the biteplate and 0.47 mm (95% CI: 0.07–1.0 mm) without the biteplate.

### 3.3 IFTD Evaluation with and without Biteplate

**FIGURE 6:**
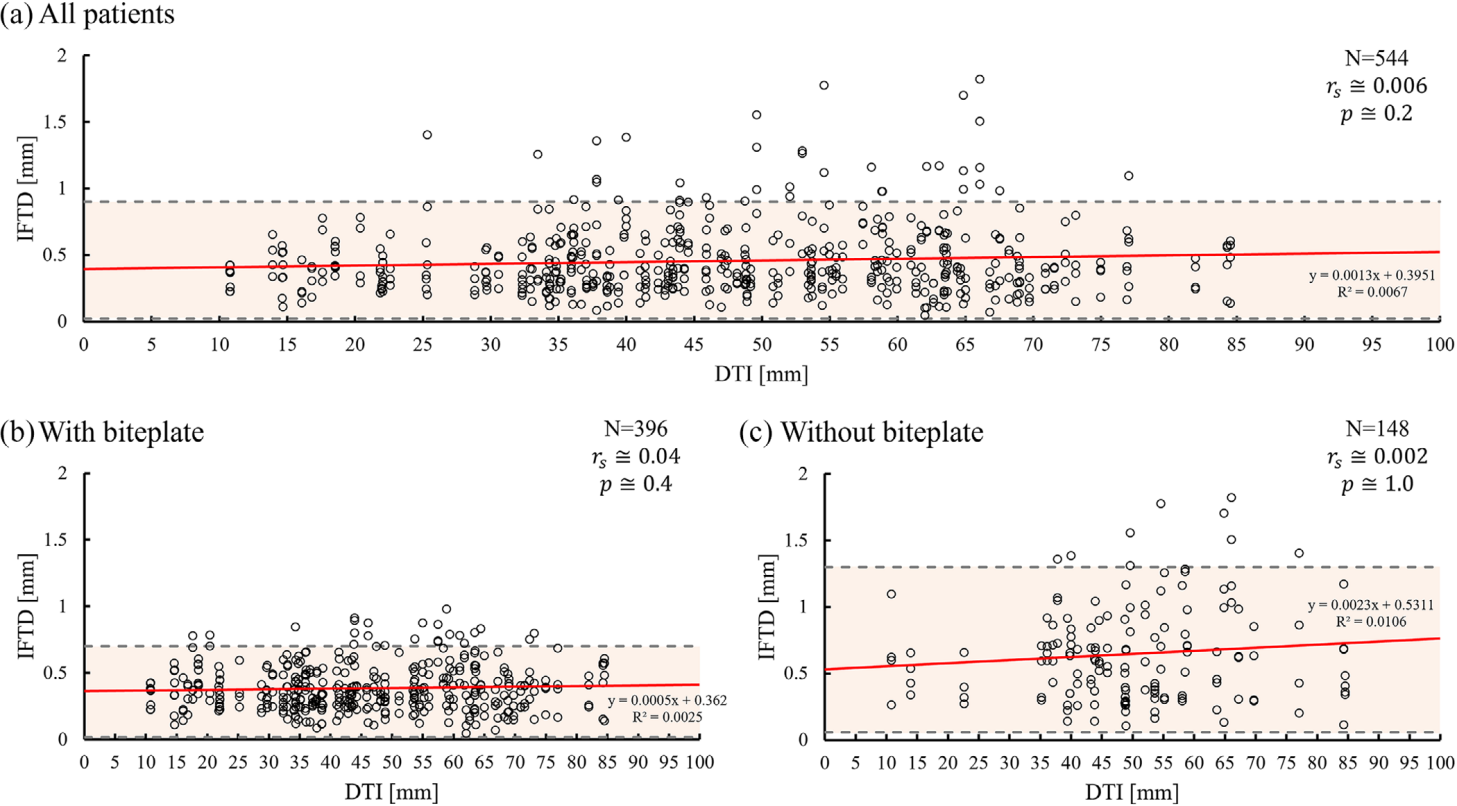
Correlation between the DTI and IFTD. (A) All patients. (B) patients with biteplate, and (C) patients without biteplate. The red line represents the linear approximation function, and the gray dotted line indicates the 95% confidence interval (CI). DTI, distance between target and isocenter; IFTD, intra‐fractional target displacement; N, number of patients; and r_s_, Spearman's rank correlation coefficient.

We obtained 544 data points from 4 fractions of 136 targets in 35 patients. Table 1 shows 396 data points from 28 patients with the biteplate and 148 data points from 7 patients without the biteplate. In evaluating IFTD, the mean IFTD was 0.39 mm (95% CI: 0.02–0.80 mm) with the biteplate and 0.65 mm (95% CI: 0.06–1.3 mm) without it. Figure 6 illustrates the correlation between DTI and IFTD for biteplate and non‐biteplate conditions. The very weak correlation observed suggests that DTI has minimal impact on IFTD, regardless of the immobilization method.

### 3.4 RD Evaluation with and without Biteplate

**FIGURE 7:**
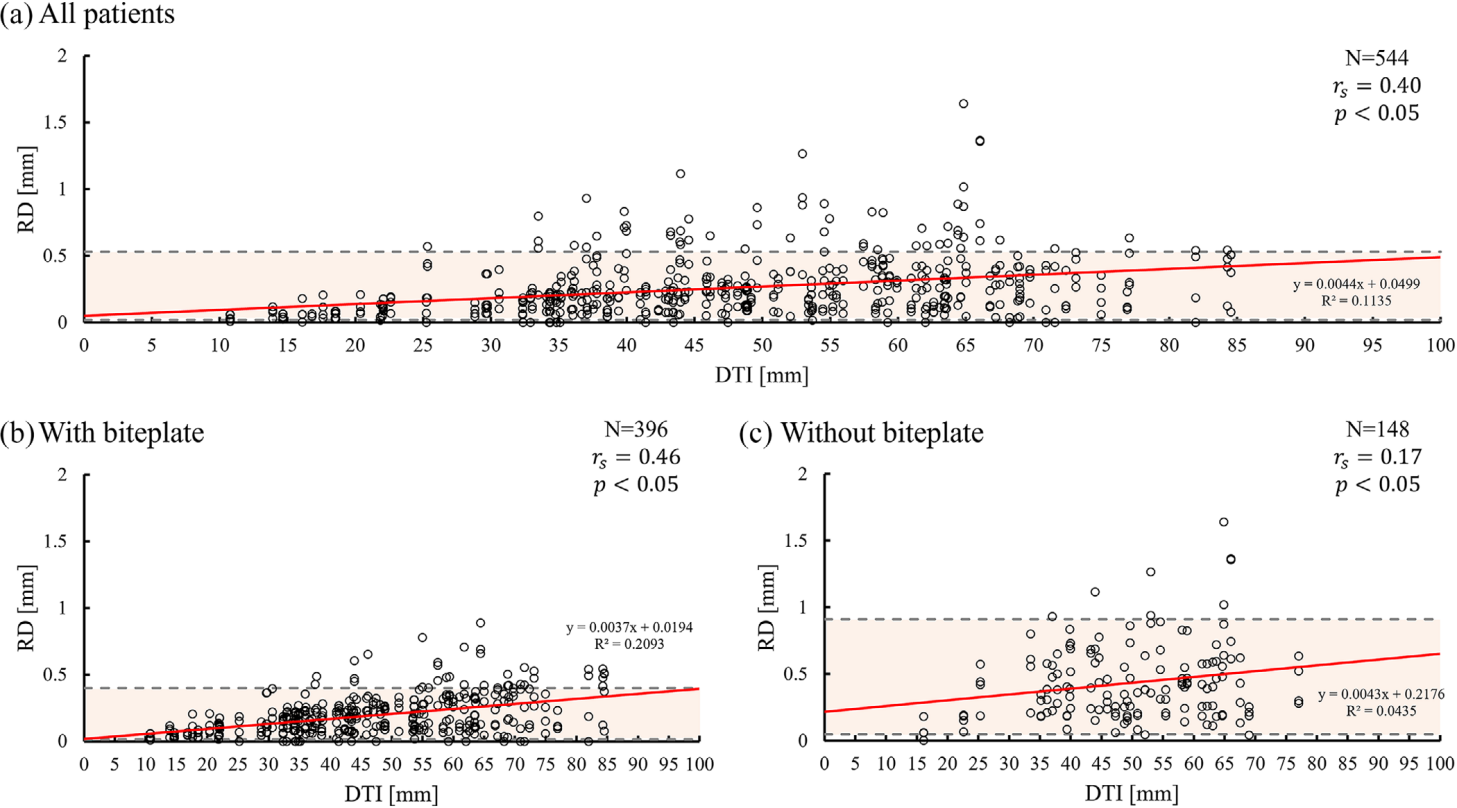
Correlation between the DTI and rotational displacement RD. (A) All patients. (B) patients with biteplate, and C) patients without biteplate. The red line represents the linear approximation function, while the gray dotted line indicates the 95% confidence interval (CI). DTI, distance between target and isocenter; RD, rotational displacement; and r_s_, Spearman's rank correlation coefficient.

**FIGURE 8:**
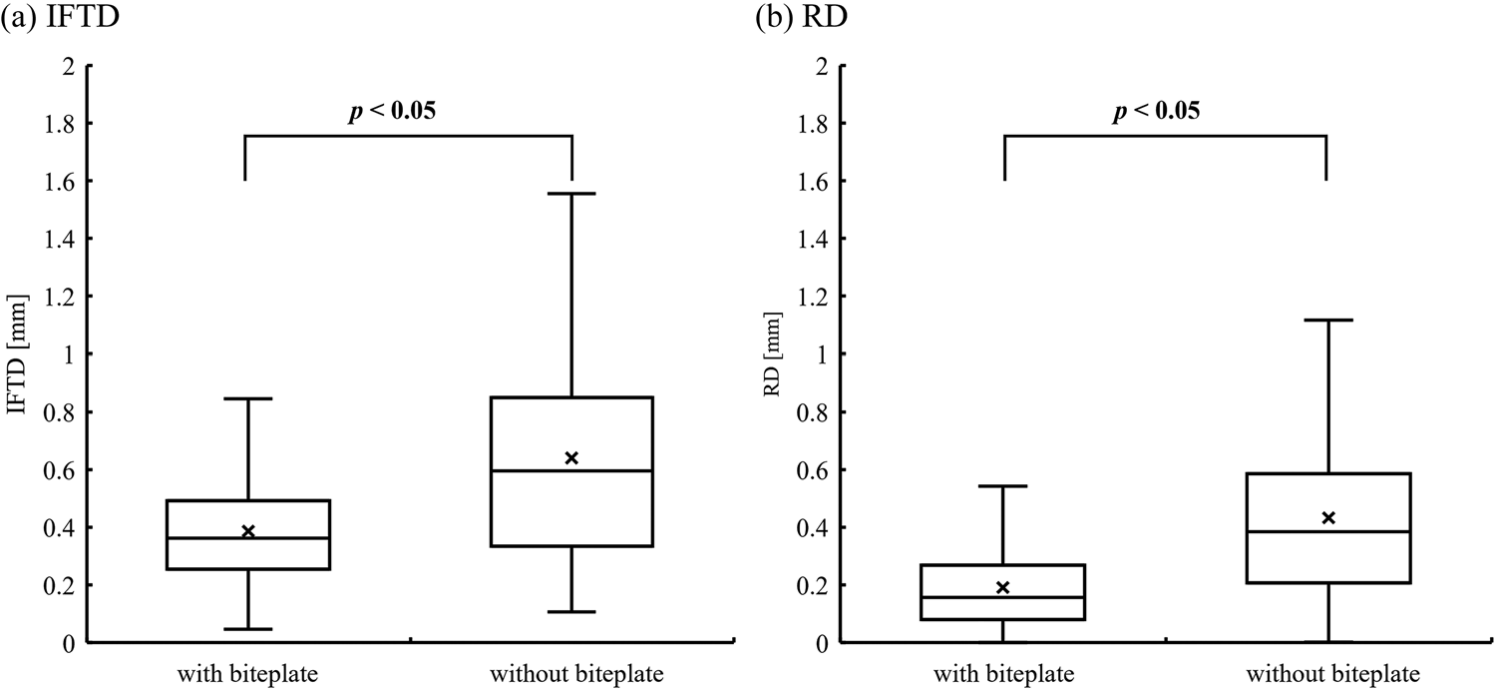
Comparison of IFTD and RD between patients with and without the biteplate. (A) IFTD. (B) RD. IFTD, intra‐fractional target displacement; RD, rotational displacement. The box represents the interquartile range (IQR), spanning from the 25th percentile to the 75th percentile. The horizontal line inside the box indicates the median (50th percentile). The whiskers extend to the smallest and largest values within 1.5 × IQR from the lower and upper quartiles, respectively. The mean value is indicated by a cross (×).

In evaluating RD, the mean RD with and without the biteplate was 0.19 and 0.43 mm (95% CI: 0.05–0.91 and 0.02–0.40 mm, respectively). As shown in Figure 7, a weak correlation was observed between DTI and RD with the biteplate, whereas a moderate correlation was found without the biteplate. This observation indicates that the biteplate may influence the relationship between DTI and RD in this study. A weak correlation was observed between DTI and RD with the biteplate, whereas a moderate correlation was found without biteplate. Figure 8 compares IFTD and RD between immobilization with and without the biteplate. Table 2 summarizes IFTD's and RD's results. Significant differences were observed between the two methods, suggesting that biteplate usage may impact IFTD and RD (p < 0.05).

**TABLE 2:** Summary of IFTD and RD.

	With biteplate						Without biteplate					
	Mean	SD	Max	95% CI			Mean	SD	Max	95% CI		
IFTD	0.39	0.17	0.98	0.02	–	0.80	0.65	0.36	1.8	0.06	–	1.3
RD	0.19	0.14	0.89	0.02	–	0.40	0.43	0.3	1.64	0.05	–	0.91

Abbreviation: IFTD, intra‐fractional target displacement; RD, rotational displacement.

### 3.5 Evaluating Couch Rotation Accuracy Using Phantom and Off‐Isocenter WL Tests

In evaluating couch motion error between CBCTs using a head phantom for each axis, the following mean errors were observed: ‐0.03 mm (max: ‐0.1 mm) AP, 0.03 mm (max: 0.1 mm) SI, 0.03 mm (max: 0.1 mm) LR, 0.03° (max: 0.1°) Yaw, 0° (max: 0) Pitch, and 0.05 (max: 0.1) Roll.

Using the off‐isocenter WL test, the maximum deviation between the BB and the radiation field center was 0.89 mm. The BB trajectory during the couch rotation formed a circular path with a walkout radius of approximately 0.61 mm. The geometric center of the BB trajectory across all couch angles deviated by 0.40 mm from the radiation field center, representing the couch rotation axis offset.

## 4 DISCUSSION

SIMT‐VMAT fSRS enables faster and more efficient treatment planning and beam delivery. Managing IFPM and ensuring accurate patient positioning is critical for successful SIMT‐VMAT fSRS implementation. In this study, we evaluated treatment time, IFPM in SIMT‐VMAT fSRS, and IFTD, quantifying the impact of IFPM on each metastasis at our facility and couch motion error.

The mean treatment time for SIMT‐VMAT fSRS in this study was approximately 14 min, which is consistent with previous reports. Clark et al. showed that SIMT‐VMAT SRS can be completed within 15 min using FFF beams at 2400 MU/min.^7^ Rami et al. reported significantly shorter treatment times for SIMT‐VMAT compared to CyberKnife (13.7 vs. 130 min).^24^ These studies confirmed the efficiency of SIMT‐fSRS; however, they do not address the relationship between treatment time and IFPM directly. In contrast, Wang et al. demonstrated that prolonged treatment times diminish positioning accuracy during stereotactic radiosurgery, suggesting increased IFPM with longer treatments.^25^ In our dataset, a very weak correlation between IFTD and treatment time was observed, which may be due to the relatively short and consistent treatment durations (∼15 min). Therefore, treatment time has a minimal impact on IFPM within this timeframe.

IFTD was calculated from pre‐ and post‐CBCT target coordinates. Agazaryan et al. stated that using a frameless thermoplastic mask system (Brainlab, Munich, Germany), the 90th percentile of translational shifts in all directions was 0.7 mm, and rotational shifts were within 0.6 degrees, with median values of 0.2 mm and 0.2 degrees, respectively.^26^ Using these findings, they concluded that a planning target volume (PTV) margin of 1.0 mm is adequate for targets clustered within 6.0 cm.^26^ Our findings complement and expand upon this by demonstrating a very weak positive correlation between DTI and IFTD. Notably, when the DTI was < 10 cm, the use of the biteplate helped maintain IFTD within 1 mm, supporting the feasibility of small margins for IFPM in clinical practice. Furthermore, IFTD significantly increased in cases without the biteplate, indicating that this immobilization aid is crucial in achieving sub‐millimeter accuracy (Figure 8).

Figures 6 and 7 show this study's most important point: the correlation between DTI and IFTD was small; however, there was a correlation between DTI and RD. Reportedly, metastases located farther from the isocenter are significantly affected by rotational components, which is consistent with our findings.^14^, ^15^, ^16^ However, the impact on metastases showed different results when the translational components were considered simultaneously. Unlike those studies that primarily examined rotational displacements in isolation, our analysis considered IFPM's translational and rotational components. Consequently, we discovered that the distance dependence of IFTD became almost negligible. Furthermore, we found that the distance between the target and the isocenter did not affect IFTD significantly. This revealed that the IFPM‐related uncertainty component of the PTV margin could be consistent across targets regardless of the distance to the isocenter.

Notably, this conclusion applies only to the IFPM component. Other uncertainty factors, including mechanical errors, couch walkout, and gantry/collimator alignment, may have different characteristics and potential distance dependencies.

Therefore, we focused on IFTD as the displacement of each target due to IFPM during SIMT fSRS. Following the limited prior research quantifying IFTD, we referred to literature on IFPM to help understand IFTD, including its contributing factors and extent. Several studies evaluated translational and rotational IFPM using commercially available immobilization systems (Table 3).^10^, ^12^, ^26^, ^27^, ^28^ For example, Tomihara et al. demonstrated that combining a thermoplastic double‐shell mask with a mouthpiece improved immobilization accuracy.^28^ This highlights the importance of immobilization design in reducing IFPM. Setup errors, especially rotational deviations, were particularly concerning in single‐isocenter stereotactic irradiation, given its higher sensitivity to rotational misalignments.^13^ Agazaryan et al. reported rotational movements within 0.6° (90th percentile) when using a frameless thermoplastic mask system, with median rotations around 0.2°, which is consistent with our findings on rotational IFPM.^26^ In contrast, frame‐based immobilization systems showed slightly different rotational error distributions, with reported maximums up to 0.87°.^29^ This finding may have reflected the differences in fixation rigidity and patient comfort. Komiyama et al. reported IFPM < 0.3 mm and 0.2° using the QFix Encompass™ system which supports non‐invasive immobilization use.^30^ These findings, alongside ours, supported the growing evidence that advanced immobilization systems can achieve the precision required for SIMT‐fSRS. Our study extended this knowledge by quantitatively evaluating IFTD during SIMT‐VMAT fSRS. Understanding IFTD remains crucial for ensuring accurate dose delivery and OAR protection as SIMT‐fSRS delivers steep dose gradients near critical structures.

**TABLE 3:** IFPM owing to the difference fixtures in previous reports.

Reference	Year	Immobilization device	*N*	IFPM (mm)	Image acquisition	Comment
Lewis BC et al.^27^	2018	Brainlab thermoplastic (head)	104	0.8 ± 0.5 (mean ± SD)	ExacTrac	not 6DOF
Babic et al.^10^	2018	CRW frame	15	0.3 ± 0.2 (mean ± SD)	CBCT	
Agazaryan et al.^26^	2021	Brainlab thermoplastic (head)	12	0.2 (median)	ExacTrac, CBCT	
Tomihara et al.^28^	2021	DSPS thermoplastic (head)	9	0.2 ± 0.1 (mean ± SD)	CBCT	
		DSPS thermoplastic (head) with mouthpiece	22	0.2 ± 0.1 (mean ± SD)	CBCT	
Ohira et al.^12^	2022	Encompass thermoplastic (head)	38	0.6 ± 0.2 (mean ± SD)	CBCT, MV	
		DSPS thermoplastic (head)	38	0.6 ± 0.3 (mean ± SD)	CBCT, MV	
Present study (ours)		Encompass thermoplastic (head) with biteplate	28	0.3 ± 0.1 (mean ± SD)	CBCT	
		Encompass thermoplastic (head) without biteplate	7	0.5 ± 0.2 (mean ± SD)	CBCT	

N: number of patients, IFPM: intra‐fractional patient motion, ExacTrac, ExacTrac Dynamic system (Brainlab AG, Munich, Germany); CBCT, cone beam computed tomography; MV, MV X‐ray imaging; Brainlab thermoplastic, a frameless SRS thermoplastic mask system (Brainlab, Munich, Germany); CRW, Cosman‐Roberts‐Wells frame (Integra‐Radionics, Burlington, MA, USA); DSPS, Double Shell Positioning System (Macromedics BV, Waddinxveen, Netherlands); Encompass, the QFix Encompass™ SRS immobilization system (CQ Medical, Avondale, PA).

We observed maximum deviations of 0.1 mm or 0.1° when evaluating couch motion error using a head phantom and CBCT at table 0°. These values were small compared with the IFPM observed in this study; however, couch rotation introduces uncertainties that must be considered when defining PTV margins. Off‐isocenter WL testing revealed a maximum deviation of 0.89 mm between the BB center and the radiation field center during couch rotation. The BB trajectory formed a circular path with a walkout radius of approximately 0.61 mm. Furthermore, this trajectory's geometric center deviated by 0.40 mm from the field center, indicating a misalignment of the couch rotation axis. Walter et al. reported that sub‐millimeter deviations following table walkout during couch rotation can cause spatial shifts in dose distribution in SIMT SRS, particularly affecting targets located far from the isocenter.^31^ Therefore, evaluation at a couch angle of 0° may underestimate uncertainties relevant to clinical dose administration. Overall, in this study, we focused on intra‐fractional patient motion at couch 0°, which can sufficiently assess patient‐related motion components; accurate PTV margin design must consider couch rotation accuracy and other mechanical uncertainties, such as gantry/collimator alignment and kV–MV isocenter congruence.

Van Herk et al. proposed a widely used margin formula accounting for systematic and random uncertainties in radiotherapy, providing a foundation for quantitative margin calculation.^32^ Subsequently, Janssen et al. extended this model by incorporating the effects of fractionation through a simulation‐based approach, demonstrating the feasibility of quantitative margin estimation when IFPM is considered.^33^ However, while this study found no dependence of IFPM on DTI, neither of these models explicitly considers the distance‐dependent characteristics of IFPM in the context of SIMT treatments. Therefore, although other factors such as mechanical and setup‐related uncertainties must be incorporated when determining PTV margins, our results contribute to a better understanding of the IFPM‐related uncertainty component relevant for margin design and may inform future efforts to individualize PTV margins for each lesion in SIMT‐fSRS.

There were some limitations in this study, one of which was poor consideration of dosimetric effects. The positional errors measured in this study may translate to an unacceptable amount of healthy brain tissue or an inadequate amount of targets. Sagawa et al. evaluated the dosimetric impact of clinical rotational setup errors in SRS for brain metastases, demonstrating that such errors for multiple brain metastases resulted in the PTV's underdosing and significant increases in V10Gy to V16Gy in LINAC‐based SRS.^34^ In addition, Yamamoto et al. compared the dosimetric parameters of LINAC‐based fSRS using single‐ and multi‐isocentric techniques, showing that the multi‐isocentric approach optimally reduced the dose to OARs and provided robustness against rotational errors in brain metastases with tumor surface distances > 6.6 cm.^35^

The second limitation is the relatively small sample size and the heterogeneous patterns of brain metastases, including size, location, and DTI variations. The median number of metastases was 3 (range: 2–13) (Table 1). Previous studies included cases with a broad range of lesion numbers, such as 1–4 by Ohira et al., 1–19 by Komiyama et al., 2–25 by Raza et al., and 20–37 by Jung et al., limiting the direct comparability of findings.^18^, ^30^, ^36^, ^37^ Therefore, caution should be exercised in generalizing our results to cases with substantially different numbers or lesion distribution. However, the overall brain structure remains relatively consistent across patients despite individual brain sizes varying up to twofold,^38^ suggesting that spatial relationships influencing target displacement are generally governed by consistent anatomical patterns. Therefore, our findings may be applicable to several clinical scenarios; nevertheless, further validation with larger and more diverse cohorts can strengthen their generalizability.

## 5 CONCLUSION

In conclusion, we evaluated target movement associated with slight patient motion during stereotactic brain radiotherapy using SIMT‐VMAT fSRS for multiple metastatic brain tumors. Our findings demonstrated that IFPM contributed a distance‐independent component to target displacement uncertainty, indicating that IFPM‐related uncertainty may not require distance‐dependent margin adjustment across multiple targets. Furthermore, the use of a biteplate reduced such motion. Other sources of uncertainty, including mechanical deviations and setup errors, should be considered separately in future investigations as this evaluation focused solely on IFPM. These findings improved our understanding of one specific margin component and may help guide the development of more individualized margin strategies in SIMT‐fSRS.

## AUTHOR CONTRIBUTIONS

Ryota Yamada carried out the data collection and wrote the first draft of this article. Ryota Yamada, Takaaki Yoshimura, and Rie Yamazaki analyzed the data. Takaaki Yoshimura performed supervision and reviewing and editing of this article. Hidefumi Aoyama supervised the project and guarantor of the data. All authors contributed to the drafting and editing of the manuscript and approved the final version.

## CONFLICT OF INTEREST STATEMENT

The authors declare that they have no conflict of interest.

## ETHICS STATEMENT

All study participants provided informed consent, and the study design was approved by the ethics committee of Hokkaido University Hospital (2205‐0017).

## Data Availability

The data that support the findings of this study are not publicly available due to [ethical/legal reasons]. However, data can be made available from the corresponding author upon reasonable request.
